# Clinical and laboratory parameters associated with febrile seizure recurrence within the first 24 h: a ten-year cohort study

**DOI:** 10.3389/fped.2024.1373848

**Published:** 2024-03-04

**Authors:** Massimo Luca Castellazzi, Adriano La Vecchia, Martina Scali, Carlo Agostoni, Giada Di Pietro, Gregorio Paolo Milani

**Affiliations:** ^1^ Pediatric Emergency Department, Fondazione IRCCS Ca’ Granda Ospedale Maggiore Policlinico, Milan, Italy; ^2^ Department of Medicine and Surgery, University of Milan-Bicocca, Monza, Italy; ^3^ Department of Clinical Sciences and Community Health, University of Milan, Milan, Italy; ^4^ Pediatric Unit, Fondazione IRCCS Ca’ Granda Ospedale Maggiore Policlinico, Milan, Italy; ^5^ Department of Clinical Sciences and Community Health, Università Degli Studi di Milano, Milan, Italy

**Keywords:** febrile seizure, recurrence, children, risk factors, fever

## Abstract

**Introduction:**

We assessed clinical and laboratory parameters associated with early recurrence of febrile seizure in patients presenting at the Emergency Department with a first episode.

**Methods:**

Case series of patients admitted to the emergency department with the first episode of febrile seizure for ten consecutive years. Exclusion criteria were focal features and prolonged duration (>15 min).

**Results:**

We included 693 patients, 284 (41%) female. Median age of 20 (IQR 15–27) months. Fifty-two (8%) patients had a recurrence within 24 h. At univariate analysis, patients with recurrent seizures had higher use of antipyretics (88% vs. 74%, *P* = 0.03, OR 2.6, 95% CI: 1.1–7.7), higher median maximal body temperature (39.3 °C, IQR 38.9–39.9, vs. 38.9, IQR 38.4–39.3, *P* < 0.001, OR 2.3, 95% CI: 1.5–2.6) and presented with a lower proportion of respiratory tract infections (54% vs. 70%, *P* = 0.02) compared to patients without recurrence. A maximal body temperature equal to or higher than 39 °C was associated with a higher recurrence (11% vs. 4%, *P* < 0.001, OR 2.9, 95% CI: 1.6–5.6). Hyponatremia was not associated with a risk of recurrence. The multivariate analysis confirmed a direct association with body temperature (OR 2.3, 95% CI: 1.5–3.7, *P* < 0.001), and an inverse association with respiratory tract infections (OR 0.4, 95% CI: 0.2–0.9, *P* = 0.01), while antipyretic use was not correlated (OR 1.9, 95% CI: 0.8–5.2, *P* = 0.2).

**Conclusions:**

High body temperature and respiratory tract infections were (directly and inversely) associated with recurrences. Consideration of these conditions might help for anticipating the probability of recurrence.

## Introduction

Febrile seizures (FS) are events affecting children between 6 months and 5 years of age, with a peak incidence between 12 and 18 months, associated with fever not associated with any infection of the central nervous system (CNS) or well-defined clinical-related causes (1–3).

They are observed in 2%–5% of children between 6 months and 5 years of age in Western Europe and the United States, being the most common neurological diseases in childhood (1–4).

FS are divided into 2 categories, that is, simple febrile seizures (SFS), primary generalized and shorter than 15 min, without altered mental status following the episode, recurrence within 24 h, and pre-existing neurologic abnormalities. If seizures are prolonged (>15 min), focal or recurrent within 24 h, with pre-existing or post-critical neurologic abnormalities they are defined as complex febrile seizures (CFS) (5–7).

Approximately 30%–40% of children with FS have a recurrence during early childhood (2, 8, 9).

Although most children with FS have only one episode during the same febrile illness, 15%–25% present a recurrence of febrile seizures (RFS) within 24 h following the first episode of febrile seizure (10). Therefore, identifying predictors of RFS could be useful in recognizing these patients and optimizing their management. Few studies have evaluated so far this issue and results are still controversial. Accordingly, predictors of RFS identified in previous studies include a low body temperature at admission to the emergency department (ED), a family history of FS, hyponatremia, seizure type, duration of the seizure, and male sex (11). On the other hand, other studies revealed that these factors were either related or unrelated (12, 13). The aim of our study is, therefore, to identify the predictors of RFS based on a retrospective analysis of clinical and laboratory data collected in our pediatric ED.

## Method

We performed a retrospective observational cohort study at a tertiary care Hospital (IRCCS Fondazione Cà Granda Ospedale Maggiore Policlinico) in Milan, Northern Italy, from, January 1st, 2012 to December, 31st, 2021. All patients aged 6 months to 5 years admitted to the ED with a first episode of FS were included. Exclusion criteria were focal features and prolonged duration (>15 min) of seizures, CNS infections, the use of anticonvulsant drugs to treat seizures, the presence of underlying diseases/conditions, such as epilepsy, chromosomal abnormalities, inborn errors of metabolism, perinatal abnormalities, delayed psychomotor development, brain tumors, intracranial hemorrhage, hydrocephalus, or a history of neurosurgery. We retrospectively collected data on demographic, clinical, and biochemical characteristics. The study was approved by The Milano Area 2 ethics committee.

Continuous data are presented as median and interquartile range and categorical data as absolute and relative frequency. To compare patients with and without recurrence of febrile seizures, the chi-square test or Fisher's exact test were used for categorical variables, and the Student's *t*-test or Mann–Whitney *U*-test for continuous ones, depending on normal or abnormal distribution tested by Shapiro Wilk test. Variables that resulted significantly associated with recurrence were then included in a multiple logistic regression model. The maximal body temperature in ED was analyzed both as continuous and categorical variable (dichotomized for < or ≥39 °C). Statistical significance was considered as a *p*-value < 0.05. Statistical analysis was performed using R software (version 3.6.3 for Windows).

## Results

We included 693 patients, 284 (41%) female and 409 (59%) male, with a median age of 20 (IQR 15–27) months. Clinical and biochemical characteristics are summarized in Table 1. Overall, 52 (8%) patients had a recurrence within 24 h after the first event. Table 2 shows the demographic, clinical, and biochemical characteristics of patients with and without recurrence. Patients with RFS reported more frequent use of antipyretics compared with patients without recurrence (88% vs. 74%, *P* = 0.03, OR 2.6, 95% CI: 1.1–7.7) and higher median maximal body temperature (39.3 °C, IQR 38.9–39.9, vs. 38.9, IQR 38.4–39.3, *P* < 0.001, OR 2.3, 95% CI: 1.5–2.6). Patients with a maximal body temperature equal to or higher than 39 °C had a rate of recurrence more than twofold compared to other patients (11% vs. 4%, *P* < 0.001, OR 2.9, 95% CI: 1.6–5.6). Patients with RFS presented a lower rate of respiratory tract infection-related symptoms (54% vs. 70%, *P* = 0.02). We did not observe other significant differences in demographic, clinical, and biochemical characteristics between patients with or without recurrence of febrile seizures, including natremia, blood glucose, and calcium levels.

**Table 1:** History, clinical and biochemical characteristics of patients.

	Number of measurements	*N* (%) or median (IQR)
Demographics		
Male	693	409 (59)
Female	693	284 (41)
Age (months)	693	20 (15–27)
History and clinical characteristics		
Positive family history of febrile seizures	693	115 (31)
Respiratory tract infection	693	474 (68)
Acute otitis media	693	14 (2)
Gastroenteritis	693	17 (2)
Post-vaccination fever	693	13 (2)
Urinary tract infection	693	3 (0.4)
Other diagnoses	693	172 (24)
Antipyretic use	693	523 (75)
Febrile seizures duration in minutes	693	2 (1–4)
Maximum fever temperature in ED, °C	693	39 (38–39)
Blood tests		
White blood cell count in mm^3^	692	13,015 (9,337–17,362)
C reactive protein, mg/dl	693	0.8 (0.3–1.7)
Sodium (direct potentiometry), mEq/L	332	132 (130–134)
Sodium (indirect potentiometry), mEq/L	362	135 (134–137)
Ionized Calcium, mEq/L	305	1.2 (1.2–1.3)
Glycemia, mg/L	693	106 (96–120)
Albumin from blood test, g/dl	360	4.5 (4.4–4.7)

ED, emergency department.

**Table 2:** Demographic, history, clinical and biochemical characteristics of patients by recurrence of febrile seizures within the first 24 h from the first event.

	Patients with RFS (*n* = 52)		Patients without RFS (*n* = 641)		
	NoM	*N* (%) or median (IQR)	NoM	N (%) or median (IQR)	*p*-value
Demographics					
Male	52	32 (62)	641	377 (59)	0.7
Female	52	20 (38)	641	264 (41)	
Age (months)	52	17.5 (14–27)	641	20 (15–27)	0.2
History and clinical characteristics					
Positive family history of febrile seizures	52	12 (41)	641	103 (32)	0.3
Respiratory tract infection	52	28 (54)	641	446 (70)	0.02
Acute otitis media	52	1 (2)	641	13 (2)	1
Gastroenteritis	52	3 (6)	641	14 (2)	0.1
Post-vaccination fever	52	1 (2)	641	12 (2)	1
Urinary tract infection	52	0	641	1 (0.5)	1
Other diagnoses	52	19 (37)	641	153 (24)	0.046
Antipyretic use	52	46 (88)	641	477 (74)	0.03
Febrile seizures duration in minutes	52	2 (1–3)	641	2 (1–4)	0.3
Maximum fever temperature, °C	52	39.3 (38.9–39.9)	641	38.9 (38.4–39.3)	<0.001
Blood tests					
White blood cell count, mm^3^	52	11,325 (9,092–15,260)	640	13,165 (9,347–17,542)	0.2
C reactive protein, mg/dl	52	1.2 (0.3–1.8)	641	1.4 (0.3–1.7)	1
Sodium (direct potentiometry), mEq/L	29	132 (129–134)	303	132 (130–134)	0.5
Sodium (indirect potentiometry), mEq/L	23	136 (134–137)	339	135 (133–137)	0.2
Ionized Calcium, mEq/L	29	1.2 (1.2–1.4)	276	1.2 (1.2–1.4)	0.9
Glycemia, mg/L	52	108 (94–122)	641	106 (96–120	0.7
Albumin, g/dl	23	4.5 (4–5)	337	4.5 (4–5)	0.3

ED, emergency department; NoM, number of measurements; RFS, recurrent febrile seizures.

After adjusting for antipyretic use and clinical presentation, body temperature remained positively correlated with a febrile seizure recurrence in the first 24 h (OR 2.3, 95% CI: 1.5–3.7, *P* < 0.001), upper respiratory tract infections, and inversely associated with RFS (OR 0.4, 95% CI: 0.2–0.9, P = 0.01). The use of antipyretics was not associated with recurrence (OR 1.9, 95% CI: 0.8–5.2, *P* = 0.2). Table 3 figures out the variables included in the logistic regression model.

**Table 3:** Independent variables included in the multiple logistic regression model and their odds ratios, 95% confidence intervals and *p*-values. The dependent variable was the recurrence of febrile seizure within 24 h from the first event.

Independent variables	OR	CI	*p*-value
Maximum fever temperature in ED, °C	2.3	1.5–3.7	<0.001
Antipyretic use	1.9	0.8–5.2	0.2
Respiratory tract infection	0.4	0.2–0.9	0.01

## Discussion

To our knowledge, this is the largest study evaluating the risk factors for a recurrence of febrile seizures within the first 24 h in a European cohort. Our findings show that patients with a maximum body temperature equal to or higher than 39 °C had higher risk of RFS than other patients. The role of fever as predictor of RFS is still a matter of debate, and different results are found in the literature. In a recent prospective study by Kubota et al. with a total of 109 children, a body temperature <39.2 °C was associated with RFS (*P* = 0.02) (10). Similar results were observed in a previous retrospective pilot study in 2020 conducted by the same group on 132 children (11). Indeed, those with a body temperature below 39 °C were more likely to experience RFS than those with higher body temperature (14). On the other hand, Jeong et al. did not find an association between body temperature and RFS (13). Moreover, there is a well-known association between a low temperature at the onset of the febrile seizure with late recurrence (after 24 h) (1). While patients with late recurrence are likely to have a low seizure threshold, our findings suggest that physicians should account for high temperature as an early recurrence trigger. We found an association between the use of antipyretics with early recurrence, that was not confirmed by the multivariate analysis. This association is likely secondary to the correlation between fever and recurrence, as it becomes not significant using a multivariate model including temperature. The relationship between antipyretic use and febrile seizure recurrence during the same febrile illness remains controversial. A recent large randomized controlled study suggested the efficacy of antipyretics compared to placebo for preventing RFS within a single event of febrile illness (15). However, a recent systematic review concluded that further studies are required to evaluate the effectiveness of antipyretics in the prevention of RFS (16).

As for natremia, we did not find any difference in sodium levels in patients with RFS and patients without recurrence. The median sodium value at venous gas analysis was 132 mEq/Lboth in patients with and without recurrence. The median value of natremia measured at laboratory analysis was 135 mEq/L in those patients without recurrence and 136 mEq/L in RFS patients. None of these results resulted statistically significant (*P* = 0.5 for venous gas analysis natremia, *P* = 0.2 for laboratory analysis). There is no consensus on the potential effect of hyponatremia on RFS. Different studies demonstrated that hyponatremia could be a predictor for recurrence. In a prospective study of 69 children, natremia was significantly lower in children with RFS than in patients without recurrence (17). Similar results were obtained in a recent retrospective study conducted by Alp et al. in which they observed that serum sodium levels were lower in children with RFS than in those without recurrence (134.20 ± 3.55 vs. 138.50 ± 2.38, *P* < 0.001) (18). A recent meta-analysis conducted by Miyagi et al. concluded that a serum sodium level lower than 134.72 mmol/L was significantly associated with RFS and it could be used as a predictor for recurrent febrile seizure (19). Nevertheless, in a large retrospective study on 315 children by Maksikharin et al. serum sodium levels were not different in children with RFS and in patients without recurrence (134.5 ± 3.2 vs. 134.9 ± 3.1, P = 0.41) (12). Similar results were obtained in the retrospective study of Navaeifar et al. (20). Differently from previous studies, we considered both blood gas analysis and laboratory analysis sodium measurement. According to the literature, sodium measured by blood gas analysis should be preferred because of its higher accuracy compared to laboratory analysis because less interfering with the hemoglobin levels and the circulating non-water fractions (albumin, immunoglobulins, clotting and non-clotting factors, lipids) (21–23).

The association between blood glucose levels and other laboratory parameters with the risk of RFS is controversial. In the retrospective study of Kubota et al., a lower blood glucose level was associated with RFS at univariate analysis (*P* = 0.047) (11). On the other hand, in another study by Kubota et al. serum glucose, C-reactive protein, and calcium levels were not different between the two groups (10). Similar results were obtained in our study.

Finally, we have found that children with RFS had a lower rate of respiratory tract infections compared to those without recurrence (*P* = 0.02). On the contrary, this result has not been observed in a retrospective study of Kubota et al. (10). Further studies are needed to better understand the correlation between clinical presentation and the risk of RFS.

Although the large sample size, our study had some limitations. At first, it is a retrospective monocentric study. Moreover, serum sodium was measured by two techniques, but data on this parameter were missing in some cases.

In a large sample of children presenting through 10 years at an Emergency Room, a high body temperature and respiratory tract infections are (directly and inversely) associated with RFS within the first 24 h. On the other hand, serum sodium, calcium, and glucose levels were not associated with a higher risk of recurrence. These findings might help for anticipating the probability of febrile seizure recurrence in childhood.

## Data Availability

The original contributions presented in the study are included in the article/Supplementary Material, further inquiries can be directed to the corresponding author.
